# Biomodification of dentin collagen with hydroalcoholic propolis extract: Impact on resin–dentin adhesion

**DOI:** 10.1111/eos.70066

**Published:** 2025-12-30

**Authors:** Zidane Hurtado Rabelo, Edinaldo Gomes de Meneses Neto, Israel Simon Andrade Costa, Juliano Sartori Mendonça, Vanara Florêncio Passos, Sérgio Lima Santiago

**Affiliations:** ^1^ Graduate Program in Dentistry Faculty of Pharmacy, Nursing and Dentistry Universidade Federal do Ceará—UFC Fortaleza Ceará Brazil; ^2^ Department of Operative Dentistry Faculty of Pharmacy, Nursing and Dentistry Universidade Federal do Ceará—UFC Fortaleza Ceará Brazil

**Keywords:** adhesives, cross‐linking reagents, dentin, propolis

## Abstract

This in vitro study evaluated the effect of hydroalcoholic propolis extract (HPE) as a collagen cross‐linking agent for preserving the dentin–adhesive interface. Dentin beams from human third molars were demineralized with 10% phosphoric acid and assigned to five treatments: distilled water (control), 6.5% grape seed extract (GSE), and HPE at 0.1%, 1%, or 10%. Elastic modulus (*n* = 10), dry mass variation (*n* = 10), and microtensile bond strength (µTBS, *n* = 8) were assessed after 24 h and after 6 months of storage. Collagen stability was analyzed using Fourier‐transform infrared spectroscopy (*n* = 10). Elastic modulus was evaluated using Kruskal–Wallis and Friedman tests, dry mass variation, and µTBS with linear mixed‐effects models. GSE produced the greatest increase in elastic modulus, followed by 1% and 0.1% HPE. After 6 months, all experimental groups maintained µTBS, except 0.1% HPE and the control, which showed reductions of approximately 30%. Spectroscopic analysis revealed increased Amide I and decreased Amide II bands in all treated groups, indicating enhanced collagen cross‐linking. Overall, HPE—particularly at 1%—improved dentin collagen stability and contributed to the preservation of the adhesive interface over time.

## INTRODUCTION

The growing demand for dental esthetics has significantly expanded the field of restorative dentistry. Influenced by social media, patients have shown increasing interest in procedures involving composite resins [1]. However, the long‐term clinical success of these restorations relies heavily on an effective adhesive protocol, which is essential for achieving durable bonding to dental tissues. Although both enamel and dentin are mineralized substrates, their adhesive behaviors differ considerably. Enamel is a relatively homogeneous tissue composed predominantly of hydroxyapatite, which allows for more predictable adhesive outcomes [2]. In contrast, dentin is a heterogeneous substrate comprising hydroxyapatite crystals, Type I collagen fibrils, and a non‐collagenous extracellular matrix rich in biological molecules such as phosphophoryn, osteocalcin, osteopontin, and osteonectin [3]. These compositional differences—along with higher water content and more complex crystal morphology—make dentin bonding substantially more challenging [4].

Type I collagen, which constitutes approximately 90% of dentin's organic matrix, plays a central role in adhesive strategies [5]. This protein has a characteristic triple‐helix structure and is rich in proline. For dentin adhesion to occur, the use of an acidic agent is required—such as an acidic primer, acidic monomers in self‐etch approaches, or phosphoric acid in etch‐and‐rinse strategies. These agents remove the mineral content and expose the collagen fibrils, allowing infiltration of resin monomers from the adhesive system [1, 6]. However, regardless of the adhesive approach, the resin–dentin interface remains susceptible to degradation over time. This degradation mainly results from incomplete infiltration of the resin into the demineralized collagen network. The resin's penetration depth is often lower than that of demineralization, primarily due to the difficulty in displacing and replacing free and weakly bound water within the interfibrillar spaces, which hinders proper hybridization [7]. Consequently, exposed collagen becomes susceptible to proteolytic degradation by endogenous enzymes such as matrix metalloproteinases and cysteine cathepsins [3].

A key goal in adhesive dentistry today is to develop strategies that prevent this degradation and enhance the long‐term mechanical performance of resin composites. In this context, collagen biomodification has emerged as a promising approach. Also referred to as collagen cross‐linking, this strategy involves the use of agents that increase intrafibrillar and interfibrillar cross‐links within the collagen matrix, thereby improving its mechanical strength and enzymatic resistance [8]. These agents are generally classified into synthetic and natural cross‐linkers, with the latter gaining attention due to their high biocompatibility, accessibility from natural sources, and lower production costs [9]. Among the natural cross‐linkers, proanthocyanidins (PACs) extracted from grape seeds (*Vitis vinifera*) have been widely studied. Their mechanism is thought to involve the interaction of phenolic groups with collagen fibrils, enhancing structural stability [10, 11]. Although their efficacy is well documented, limitations such as interface pigmentation remain under investigation [12, 13].

As an alternative to PACs, propolis extract has recently drawn interest. Propolis is a resinous substance collected by *Apis mellifera* bees and used to seal hives and protect against intruders. During collection, bees mix plant resins with wax and β‐glucosidase enzymes from their saliva, leading to the hydrolysis of glycosylated flavonoids into biologically active aglycone flavonoids [14]. The use of propolis extract in dentistry has already been reported across several areas, highlighting its therapeutic potential [15]. In particular, Brazilian propolis extract is known for its chemical complexity, containing high concentrations of flavonoids, phenolic compounds, terpenoids, aromatic acids, vitamins, amino acids, and trace minerals [16]. These characteristics can promote collagen cross‐linking through hydrogen and covalent bonding, thereby enhancing the mechanical stability and enzymatic resistance of the hybrid layer. These interactions are proposed to reduce collagen degradation and improve resin–dentin bond durability. This unique composition is believed to underpin its broad spectrum of biological activities, reinforcing its potential as a multifunctional agent in restorative dentistry.

Although propolis extract has been previously investigated for its potential to enhance dentin adhesion, to the best of our knowledge, its hydroalcoholic extract has not yet been evaluated as a dentin pretreatment in adhesive procedures. Therefore, the aim of this study was to evaluate the effect of hydroalcoholic propolis extract (HPE) as a collagen biomodifier on the preservation of the resin–dentin adhesive interface. The null hypothesis tested was that HPE, regardless of concentration, would have no effect on dentin collagen stability or on the long‐term bond strength of resin–dentin interfaces.

## MATERIAL AND METHODS

This study was approved by the Research Ethics Committee of Federal University of Ceará (Approval #5.235.288). In this in vitro laboratory investigation, the following dentin treatments were evaluated: (i) distilled water (technique control—TC); (ii) grape seed extract (GSE) at 6.5% (positive control); (iii) HPE at 0.1%; (iv) HPE at 1%; and (v) HPE at 10%. To assess the biomodification potential of the solutions, three‐point bending tests (*n* = 10 specimens/treatment) and dry mass measurements (*n* = 10 specimens/treatment) were performed using a universal testing machine and a precision analytical balance, respectively. Additionally, qualitative analysis was conducted using Fourier‐transform infrared spectroscopy (FT‐IR) (*n* = 10 specimens/treatment). To evaluate the influence on the adhesive procedure, a microtensile bond strength (µTBS) test (*n* = 8 specimens/treatment) was performed after 24 h and following 6 months of aging.

### Specimen preparation

For this study, 20 sound human third molars, indicated for extraction for reasons unrelated to this research, were collected. All patients gave written consent to their use for research. The teeth were cleaned and stored in 0.1% thymol solution for a maximum period of 3 months. For the elastic modulus, dry mass measurement, and FT‐IR tests, mid‐coronal dentin discs were obtained using a metallographic cutter (Isomet 1000, Buehler). The enamel at the margins was removed, and approximately three dentin beams (1.7 × 0.5 × 6 mm^3^) were obtained from each tooth, resulting in ten specimens per treatment. These beams (control and tested groups) were immersed in a 10% phosphoric acid solution in a beaker for a period of 5 h at room temperature under constant agitation using a magnetic stirrer [17]. Afterward, specimens were thoroughly rinsed with distilled water, and the absence of residual mineral phase was confirmed by the lack of phosphate peaks in the FT‐IR spectra.

### Preparation of immersion solutions

For the experimental treatments, solutions were prepared from a concentrated hydroalcoholic red Brazilian propolis extract obtained from a compounding pharmacy. The extract contained 60% ± 20% alcohol, with a raw material ratio of 1:20 and a density of 0.900 ± 0.150 g/mL (nominal 0.89 g/mL). It was diluted in an ethanol/water (1:1, v/v) mixture to obtain final concentrations of 0.1%, 1%, and 10% (v/v). For positive control, dry GSE (*V. vinifera*, Jarrow Formulas; 95% polyphenols) was used to prepare an ethanol/water (1:1) solution at a concentration of 6.5% (w/v). The resulting solution was centrifuged for 1 h at room temperature, and the supernatant was used for the subsequent tests.

### Sample size calculation

The sample size for each test was estimated based on mean values and standard deviations reported in previous studies with similar experimental designs and outcomes. A minimum of *n* = 10 specimens per group was used for modulus of elasticity and dry mass variation assessment [18], and *n* = 8 teeth (experimental unit) per group for µTBS testing [19]. These numbers were selected to achieve a statistical power of at least 80% (*β* = 0.20) at a significance level of *α* = 0.05 considering the variability observed in the literature. Specifically, for modulus of elasticity, 10 specimens per group were required to detect a 50% difference between groups (baseline mean of 5.5 and standard deviation of 2.2). For µTBS, at least six teeth per group were required to detect a 30% difference between groups (mean of 22 MPa and standard deviation of 4.5). The sample size was increased to eight teeth per group to compensate for potential specimen loss during preparation and testing.

### Elastic modulus

This assessment was performed using a three‐point bending test. A universal testing machine (Instron 3345, Instron) with a 5.0 N load cell and a crosshead speed of 0.5 mm/min was employed. Each specimen was properly positioned with its long axis perpendicular to the supporting rods, with a span length of 4.0 mm. Elastic modulus was converted into MPa according to the formula:

*E* = *F* × *L* ^3^/4 × *b* × *h* ^3^ × *f*

 where *F* is the maximum load (N), *L* is the support span (mm), *b* is the width of the specimen (mm), *h* is the thickness of the specimen (mm), and *f* is the displacement (mm). After obtaining the baseline values, specimens were randomly distributed into different groups using microsoft excel 2016 (Microsoft). Next, the specimens were immersed in their respective biomodification solutions for 1 h, rinsed with distilled water for 30 s, and the elastic modulus was reassessed [8]. To verify the stability of the cross‐links, specimens were stored in saliva‐like buffer solution (1.5 mM CaCl_2_, 0.9 mM NaH_2_PO_4_, 0.13 M KCl, and 5 mM NaN_3_, buffered at pH 7.0) at 37°C without agitation. After 7 and 14 days, measurements of the elastic modulus were repeated on the same specimens, with the storage solution being replaced daily.

### Dry mass variation

To evaluate dry mass gain after biomodification, a five‐decimal‐place precision analytical balance (accuracy: 0.01 mg, AUX‐220, Shimadzu) was used. The same beams used for elastic modulus measurement were employed for this analysis. Initially, the specimens were stored in a vacuum desiccator containing colloidal silica for 24 h at room temperature to remove moisture and achieve complete dehydration, enabling the weighing of isolated collagen. Subsequently, the specimens were immersed for 1 h in their respective biomodification solutions, returned to the vacuum desiccator for another 24 h, and weighed again to determine post‐biomodification mass. Dehydration and weighing of the specimens were repeated, and the specimens were also evaluated after 7 and 14 days of storage [8].

### Fourier‐transform infrared spectroscopy (FT‐IR)

Specimens (1.7 × 0.5 × 6 mm^3^) used for FT‐IR analysis (*n* = 10/treatment) were demineralized for 5 h and subsequently immersed for 1 h in their respective biomodification solutions, as previously described. After being placed in a vacuum desiccator for 24 h, the demineralized and biomodified collagen beams were ground using a mortar and pestle. FT‐IR analyses were conducted using an IRTracer‐100 spectrometer (Shimadzu) in the infrared range of 4000–400 cm^−1^ using KBr pellets, with 64 scans per sample and a resolution of 4 cm^−1^. The analysis focused on Amide I (∼1650 cm^−1^), Amide II (∼1550 cm^−1^), and Amide III (∼1240 cm^−1^) bands, which represent characteristic collagen vibrations. Each chemical reading was assessed in triplicate, thus generating three absorption spectra for each specimen.

### Adhesive procedure

Forty sound human third molars, extracted for clinical reasons unrelated to this study, were sectioned 3 mm above and below the cementoenamel junction using a low‐speed diamond disc mounted in a sectioning machine (Isomet 4000; Buehler) until mid‐coronal dentin was exposed. The surfaces were then polished with 600‐grit silicon carbide paper under water irrigation for 30 s to create a standardized smear layer. The specimens were examined under a stereomicroscope (Leica S8APO, Model MEB 115) to exclude the presence of enamel or pulp chamber perforations. Dentin surfaces were etched with 37% phosphoric acid (Condac 37%, FGM) for 15 s, followed by rinsing with distilled water for twice that time and blot drying with absorbent paper.

The biomodification solutions were actively applied to the dentin surface using a microbrush for 60 s. Excess moisture was removed with absorbent paper, and the adhesive system (Adper Single Bond 2, 3 M ESPE) was actively applied in two 15‐s layers, followed by gentle air‐drying for 5 s. Light‐curing was performed for 10 s using a Grand Valo Cordless curing unit with an intensity of 1200 mW/cm^2^ (Ultradent do Brasil). A resin composite build‐up (Filtek Z350 XT, 3 M ESPE) was created in 1 mm increments up to a total height of approximately 5 mm, with each layer light‐cured for 20 s [17].

### Microtensile bond strength (µTBS)

Resin–dentin sticks with an approximate cross‐sectional area of 1 mm^2^ were obtained by sectioning the restored teeth (*n* = 8/treatment). Specimens were stored in distilled water (pH = 7) in an incubator at 37°C for 24 h before testing. A caliper was used to measure the exact cross‐sectional area of each specimen. Approximately half of the specimens from each tooth were evaluated immediately, whereas the remaining samples were stored for 6 months for aging, under the same conditions, with weekly storage solution changes.

The specimens were fixed to a Geraldelli jig device using a cyanoacrylate‐based adhesive (Super Bonder Gel, Loctite) and attached to a universal testing machine (Instron 3345, Instron), where they were loaded in tension until failure using a 500 N load cell at a crosshead speed of 0.5 mm/min.

The fractured specimens were examined under a stereomicroscope at 80× magnification (Leica S8APO, Model MEB 115) to classify the failure mode as adhesive, cohesive in resin, cohesive in dentin, or mixed—in which two modes of failure co‐occurred.

### Statistical analysis

After data collection, the results were tabulated and statistically analyzed using jamovi 2.3.38. Shapiro–Wilk test was applied to assess data normality. Data from elastic modulus failed in normality test and were analyzed by Kruskal–Wallis and Friedman tests.

Dry mass and µTBS (MPa) were analyzed using linear mixed‐effects models (LMMs) to account for the repeated‐measures structure of the data. For each outcome, the corresponding measurement was treated as the dependent variable. Fixed effects included the dentin pretreatment solution (five levels, with distilled water as the reference), storage time (four levels for dry mass analyses and two levels for bond strength analyses, with the baseline measurement used as the reference), and their interaction; all fixed factors were coded as dummy variables. Time was modeled as the within‐subject (repeated‐measures) factor. A random intercept for each specimen (dry mass) or each tooth (µTBS) was included to account for within‐unit dependence.

Model parameters were estimated using restricted maximum likelihood, and degrees of freedom were approximated using the Satterthwaite method. Model fit was assessed with Akaike's Information Criterion and marginal and conditional *R* ^2^ values. When applicable, Bonferroni‐adjusted post hoc pairwise comparisons were performed. Model assumptions were verified using the Kolmogorov–Smirnov and Shapiro–Wilk tests, complemented by inspection of *Q*–*Q* diagnostic plots. Statistical significance was set at *p* < 0.05. FT‐IR data were qualitatively analyzed to assess the formation of chemical bonds.

## RESULTS

Regarding the elastic modulus results, the 6.5% GSE treatment showed a statistically significantly higher elastic modulus at the immediate evaluation than seen for all other treatments (*p* < 0.05). The control and HPE 10% groups did not differ from each other. After 7 days, the control group differed statistically significantly from the GSE and HPE 10% groups. After 7 and 14 days of storage, however, no significant differences from baseline were observed, and the values returned to their initial levels (Table 1).

**TABLE 1:** Mean (SD) values of the elastic modulus, in MPa, of dentin beams immersed in different biomodification solutions at different time points of the experiment (*n* = 10 per treatment).

		After biomodification treatment		
Treatment	Before treatment	Immediate	7 days	14 days
Distilled water	1.2 (0.7) Aa	1.8 (2.1) Aa	0.5 (0.1) Ba	1.7 (1.5) Aa
Grape seed extract 6.5%	1.4 (0.3) Aa	9.7 (3.3) Cb	1.2 (0.4) Aa	1.1 (1.0) Aa
Hydroalcoholic propolis extract 0.1%	0.9 (0.4) Aa	3.8 (2.3) Bb	0.7 (0.3) ABa	0.7 (0.5) Aa
Hydroalcoholic propolis extract 1%	1.1 (0.4) Aa	4.5 (2.7) Bb	0.8 (0.4) ABa	0.6 (0.6) Aa
Hydroalcoholic propolis extract 10%	1.6 (0.6) Aa	2.8 (1.3) Aa	1.2 (0.8) Aa	1.7 (1.9) Aa

*Note*: Different capital letters indicate statistical differences in column (*p* < 0.05). Different lowercase letters indicate statistical differences in rows (*p* < 0.05).

For dry mass, the LMM showed that fixed effects explained 12.6% of the variance (marginal *R* ^2^), whereas the full model accounted for 93.7% (conditional *R* ^2^). The intraclass correlation coefficient was high (ICC = 0.928), indicating substantial between‐specimen variability. Storage time had a significant effect on dry mass, whereas pretreatment solution did not (Table 2). A significant interaction between pretreatment and storage time was detected, suggesting that temporal changes varied across solutions. However, the only significant fixed‐effect contrast for storage time was a small decrease from baseline to 14 days (*β* = −1.55 × 10^−4^; 95% CI: −2.97 × 10^−4^ to −0.13 × 10^−4^). No pairwise comparisons within the interaction remained significant after Bonferroni adjustment (*p* > 0.05), indicating that although the interaction was significant at the omnibus level, individual comparisons did not survive multiplicity correction (Table 2).

**TABLE 2:** Mixed‐effects dummy variable linear regression analysis of the dry mass variation (×10^−4^ g) as a function of the pretreatment solutions and time of assessment.

Independent variable		*β*	95% CI for *β*
Treatment	Level		
Constant	Distilled water before treatment	31.9	28.1–35.6
Biomodification	Distilled water	Reference	–
	Grape seed extract 6.5%	2.23	−3.09 to 7.55
	Hydroalcoholic propolis 0.1%	−0.38	−5.70 to 4.94
	Hydroalcoholic propolis 1%	−3.97	−9.29 to 1.35
	Hydroalcoholic propolis 10%	−2.10	−7.42 to 3.22
Time	Before treatment	Reference	–
	Immediately after treatment	−0.34	−1.76 to 1.08
	7 days	−0.43	−1.85 to 0.99
	14 days	−1.55	−2.97 to −0.13
Interactions: biomodification × time	Distilled water before treatment	Reference	–
	GSE 6.5% × immediately after treatment	0.88	−1.13 to 2.89
	HPE 0.1% × immediately after treatment	−0.51	−2.52 to 1.50
	HPE 1% × immediately after treatment	−1.88	−3.89 to 0.13
	HPE 10% × immediately after treatment	−1.00	−3.01 to 1.01
	GSE × 7 days	0.10	−1.91 to 2.11
	HPE 0.1% × 7 days	−0.32	−2.33 to 1.69
	HPE 1% × 7 days	0.94	−1.07 to 2.95
	HPE 10% × 7 days	0.90	−1.11 to 2.91
	GSE × 14 days	0.90	−1.11 to 2.91
	HPE 0.1% × 14 days	1.15	−0.86 to 3.16
	HPE 1% × 14 days	1.51	−0.50 to 3.52
	HPE 10% × 14 days	0.18	−1.83 to 2.19

Abbreviations: GSE, grape seed extract; HPE, hydroalcoholic propolis extract.

**FIGURE 1:**
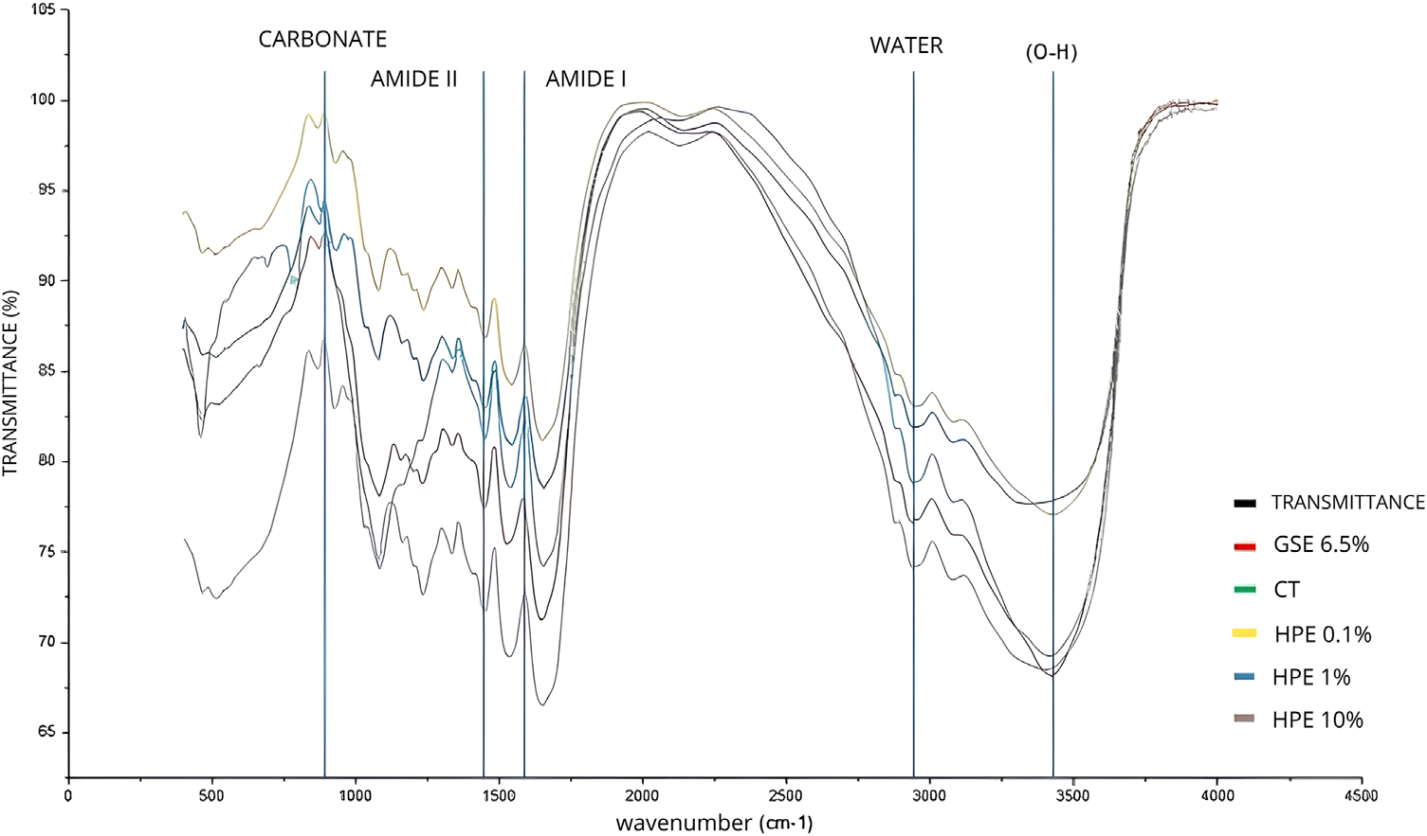
Fourier transform infrared spectroscopy (FT‐IR) of collagen beams after treatment with the respective tested solutions. CT, control (distilled water); GSE, grape seed extract; HPE, hydroalcoholic propolis extract.

When analyzing the FT‐IR spectra (Figure 1), it was observed that nearly all groups showed an increase in the peaks corresponding to Amide I (∼1680–1600 cm^−1^). A decrease in the peaks related to Amide II (∼1580–1480 cm^−1^) was also noted; however, this decrease was less pronounced in the exhibited a significantly smaller decrease in the water peak (∼3793–2652 cm^−1^) compared to the others. A reduction in the peak located between 3200 and 3600 cm^−1^ was also observed, indicating a decrease in the number of free hydroxyl (O–H) groups, which was slightly less marked only in the HPE 1% group. Finally, the CT group showed a reduction in the carbonate peaks located at approximately ∼875 cm^−1^ (Figure 1).

For µTBS (MPa), the model showed that the fixed effects accounted for 28.2% of the variance (marginal *R* ^2^ = 0.282), and the full model explained 60.8% (conditional *R* ^2^ = 0.608) (Table 3). The intraclass correlation coefficient was moderate (ICC = 0.454), indicating substantial variance attributable to differences among teeth. Storage time showed a significant main effect, whereas neither pretreatment solution nor the pretreatment × storage time interaction reached significance. The parameter estimate for storage time (6 months vs. immediate) indicated a markedly lower bond strength (*β* = −10.48; 95% CI: −16.51 to −4.44) after 6 months.

**TABLE 3:** Mixed‐effects dummy variable linear regression analysis of the microtensile bond strength (MPa) as a function of the pretreatment solutions and storage time.

Independent variable		*β*	95% CI for *β*
Treatment	Level		
Constant	Distilled water at immediate period	38.90	33.13 to 44.68
Biomodification	Distilled water	Reference	–
	Grape seed extract 6.5%	−0.133	−8.30 to 8.03
	Hydroalcoholic propolis 0.1%	6.673	−1.49 to 14.84
	Hydroalcoholic propolis 1%	3.901	−4.26 to 12.06
	Hydroalcoholic propolis 10%	2.728	−5.44 to 10.89
Time	Immediate	Reference	to
	6 months	−10.478	−16.51 to −4.44

**FIGURE 2:**
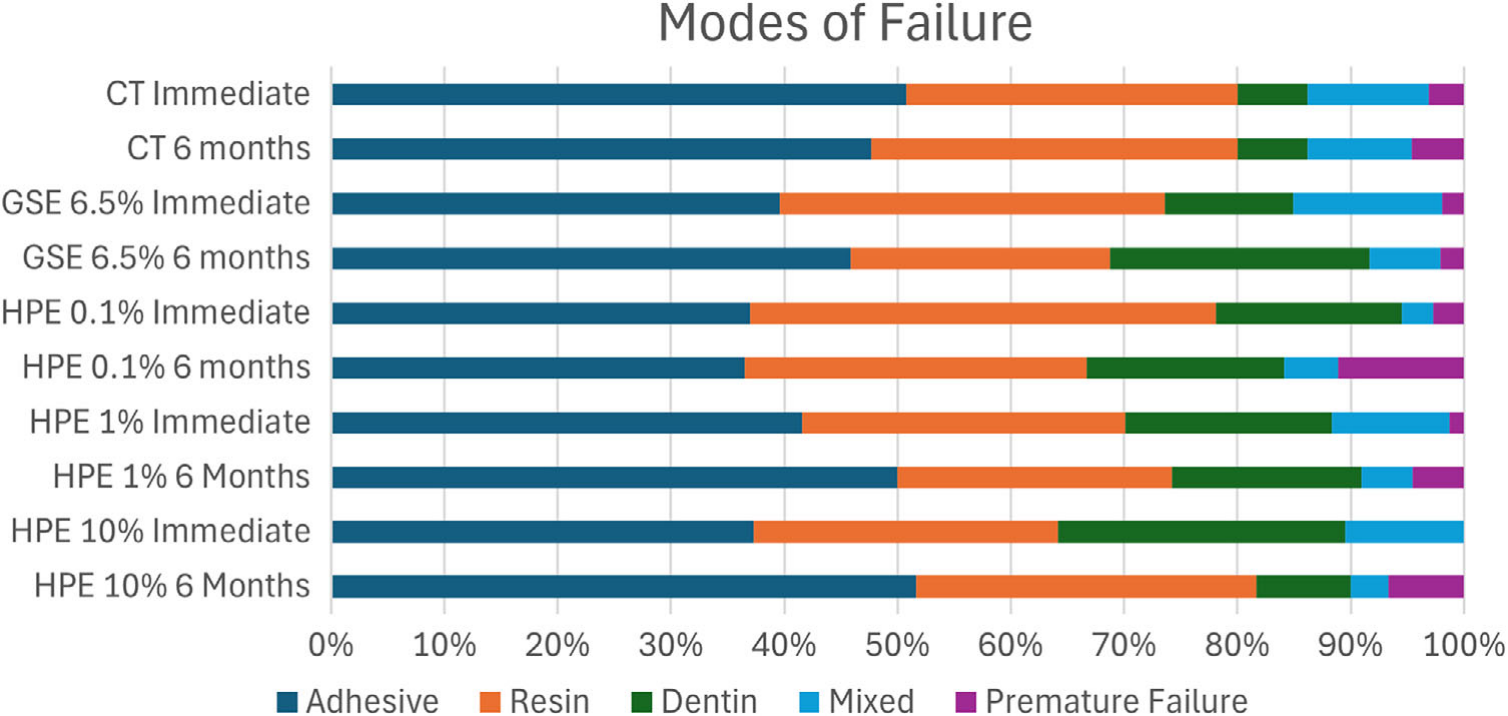
Relative frequency (%) of failure modes in dentin specimens from each group, tested immediately and after 6 months of storage. CT, control (distilled water); GSE, grape seed extract; HPE, hydroalcoholic propolis extract.

The distribution of failure modes for the debonded specimens (%) is presented in Figure 2. Adhesive failure was the predominant mode in all groups, except in the HPE 0.1% group tested immediately.

## DISCUSSION

Despite the wide range of dentin collagen biomodifiers that have been investigated, failures at the adhesive interface remain common. These failures compromise the longevity of esthetic restorations and increase treatment costs in both private and public healthcare systems [20]. Therefore, the search for cost‐effective strategies to enhance adhesive performance and promote clinical efficiency continues. Natural agents derived from renewable and readily available sources have emerged as promising alternatives, being generally safe, accessible, and affordable [1, 5, 10]. These natural compounds, particularly those rich in phenolic groups, can interact with the amino acid residues of type I collagen, promoting nonenzymatic cross‐linking and improving the structural integrity of the hybrid layer [10].

Although the GSE used in this study contains PACs—the gold standard among natural biomodifiers widely documented in the literature—GSE has a notable limitation: It can stain dental tissues, which restricts its use in esthetic areas [4, 10, 13, 21]. In response, recent studies have focused on identifying new biocompatible biomodifying agents with fewer esthetic drawbacks [17, 19, 22]. In this context, HPE emerges as a potential alternative, as it contains flavonoids and phenolic acids capable of promoting similar cross‐linking effects without compromising esthetic appearance [16].

In the current study, the 6.5% GSE treatment with a 1‐h application time statistically significantly enhanced the biomechanical properties of dentin collagen, particularly evident in the immediate increase in the elastic modulus. These results are consistent with prior research reporting increased elastic modulus following GSE treatment [22]. This increase in elastic modulus can be attributed to the formation of additional intermolecular cross‐links between collagen fibrils mediated by the phenolic compounds in the propolis extract. These bonds reduce molecular mobility and water content within the matrix, leading to greater stiffness and mechanical stability of demineralized dentin. Additionally, it has been suggested that diluting GSE in water rather than ethanol may further improve its effectiveness [8, 21, 23].

In comparison, HPE at 0.1% and 1% concentrations also increased elastic modulus in the immediate evaluation, although to a lesser extent than GSE. Although the three‐point bending test is widely used for evaluating the elastic modulus of demineralized dentin, an inherent limitation of this method is that repeated measurements on the same specimen may introduce mechanical fatigue or microdamage accumulation over time. Even though the applied load was restricted to the linear elastic region, as conventionally adopted in dentin biomechanics studies, it cannot be fully guaranteed that the repetition of bending cycles did not alter the structural integrity of the beams. Therefore, part of the variation observed across time points may reflect not only the effects of biomodification but also potential fatigue‐related changes induced by repeated mechanical loading. This limitation should be considered when interpreting the temporal effects, and future studies using independent specimens for each evaluation period or alternative nondestructive mechanical tests are recommended to confirm these findings.

Interestingly, even at 10%, HPE produced considerably less staining during handling, indicating a potential esthetic advantage over GSE. The increasing importance of esthetics in modern dentistry reflects patient pursuit of improved appearance and self‐confidence, which can influence their social interactions. Thus, HPE appears to be a promising alternative to PACs‐rich agents, with a lower risk of compromising esthetic outcomes. Nonetheless, further studies are needed to assess its color stability.

Regarding mass variation, no statistically significant differences were found among groups and time of evaluations. The slight number increase in mass may reflect the incorporation of phenolic compounds into the collagen matrix or reduced hydrolytic loss, suggesting enhanced molecular stabilization. The GSE positive control showed an increase only in the immediate assessment (3.46 ± 7.21) and returned to baseline values thereafter. The observed variability may be attributed to the large specimen size, which could influence the rate of collagen degradation. Importantly, the use of three HPE concentrations aimed to identify the lowest pharmacologically effective dose. However, even the 10% concentration did not show benefits, suggesting that lower concentrations may still be effective.

The use of FT‐IR is linked to the behavior of collagen molecules in dentin adhesion. Thus, its application has become increasingly common when investigating changes in collagen cross‐linking processes [24]. In this study, the most relevant absorption peaks were identified as Amide I (∼1650 cm^−1^), Amide II (∼1560 cm^−1^), water peak, carbonate peak, and the free hydroxyl (O–H) peak (3200–3600 cm^−1^). The first two are intrinsically associated with collagen cross‐link formation, where the Amide II absorption peak is expected to be stronger, whereas Amide I peak should remain unchanged, indicating the number of bonds formed during this mechanism [25]. These spectral modifications indicate the formation of additional hydrogen and covalent cross‐links within the collagen fibrils, which is consistent with the mechanical stiffening observed in the elastic modulus results. The relative content of the carbonate peak reflects the extent of carbonate incorporation into the hydroxyapatite structure. This compound is highly prevalent in adhesive systems and is calculated by the ratio between the carbonate ν2 band area (840–890 cm^−1^) and the phosphate v1v3 band area (900–1180 cm^−1^) [26, 27].

The presence of water plays a significant role in the properties of mineralized tissues, such as crystallographic parameters and color—key characteristics, especially for dental tissues [28]. It has been demonstrated that the ratio between the water band areas (3793–2652 cm^−1^) and the phosphate v1v3 band (900–1180 cm^−1^) is related to water concentration in the samples [29]. When evaluating the FT‐IR spectra, all groups showed an increase in peaks related to Amide I. However, a decrease in peaks associated with Amide II was observed, which was lower in the HPE 0.1% and HPE 1% groups. Another critical finding of this study relates to the water peak absorbance in the tested groups, where all demonstrated a reduction. However, the HPE 0.1% and 1% groups showed a significantly smaller decline compared to the others. Additionally, a decrease in the free hydroxyl (O–H) peak was noted. These two findings indicate reduced water incorporation into the specimens and are linked to the closer packing of triple‐helix chain molecules, which prevents water accumulation in this region. With lower water content, collagen degradation is expected to decrease, resulting in greater stability of the formed bonds [30]. Finally, the distilled water treatment resulted in exhibited reduction in carbonate peaks, indicating that both the test groups and the group treated with GSE 6.5% are superior in terms of interaction with the components of adhesive systems. Overall, the spectroscopic evidence supports the mechanical and adhesive findings, indicating that molecular cross‐linking promoted by HPE translated into improved structural and adhesive stability.

Bond strength data revealed that the 1% and 10% HPE groups maintained stability after 6 months, whereas the 0.1% group did not (31.7 ± 0.03 MPa). The elastic modulus and dry mass variation results serve as surrogate markers of collagen cross‐linking and matrix stabilization, reflecting the potential of biomodifiers to resist enzymatic and hydrolytic degradation and preserve long‐term bond strength. In the current study, the 1% HPE concentration consistently promoted enhanced mechanical and mass stability, as well as superior adhesive performance after aging. Conversely, 0.1% HPE produced an initial increase in elastic modulus but failed to sustain bond strength over time, possibly due to insufficient cross‐link density or weaker molecular interactions with the collagen matrix. However, future studies should evaluate potential cytotoxic effects at this concentration. Based on the present results, the null hypothesis must be rejected. The findings demonstrate that the HPE influences dentin collagen stability and resin–dentin bond strength over time.

Recent studies have proposed incorporating natural biomodifiers directly into adhesives or acid conditioners to simplify clinical protocols and minimize laboratory variability [17, 31, 32]. For instance, incorporating epigallocatechin gallate into adhesive systems reduced gelatinolytic activity within the hybrid layer without affecting bond strength [31]. Similarly, the incorporation of Brazilian red propolis into an experimental adhesive resulted in superior sealing ability and performance compared to commercial products [32, 33]. Based on this evidence, integrating HPE into adhesive formulations may yield even more favorable outcomes.

One limitation of this study is the exclusive use of sound dentin. In clinical practice, however, most adhesive procedures involve carious or non‐carious cervical lesions. Therefore, future investigations should include caries‐affected dentin and incorporate additional laboratory analyses, such as in situ degree of conversion, nanoleakage assessment, and comprehensive color evaluation to determine esthetic compatibility. The present study also shares limitations inherent to in vitro designs, which may not fully reproduce the complexity of clinical conditions, including saliva dynamics, occlusal stresses, and patient‐related factors. Moreover, the relatively short storage period restricts the ability to predict the long‐term performance of the biomodified interfaces. Hence, studies involving extended aging and in vivo models are necessary to validate these findings and assess their clinical relevance. Within the limitations of this in vitro study, the use of HPE had a positive effect on dentin collagen stability and resin–dentin bond strength. Among the tested concentrations, 1% HPE yielded the most favorable results, enhancing both mechanical and spectroscopic outcomes. Overall, these findings highlight the potential of HPE as a natural strategy to improve the longevity of adhesive interfaces.

## AUTHOR CONTRIBUTIONS

**Conceptualization**: Zidane Hurtado Rabelo, Sérgio Lima Santiago. **Formal analysis**: Juliano Sartori Mendonça, Vanara Florêncio Passos. **Methodology**: Zidane Hurtado Rabelo. **Validation**: Edinaldo Gomes de Meneses Neto, Israel Simon Andrade Costa. **Visualization**: Edinaldo Gomes de Meneses Neto, Israel Simon Andrade Costa. **Writing—original draft**: Zidane Hurtado Rabelo, Edinaldo Gomes de Meneses Neto, Israel Simon Andrade Costa. **Writing—review and editing**: Sérgio Lima Santiago, Vanara Florêncio Passos. **Resources**: Sérgio Lima Santiago. **Supervision**: Vanara Florêncio Passos, Sérgio Lima Santiago.

## CONFLICT OF INTEREST STATEMENT

The authors declare no conflicts of interest.
